# Suppressive effects of a transient receptor potential melastatin 8 (TRPM8) agonist on hyperthermia-induced febrile seizures in infant mice

**DOI:** 10.3389/fphar.2023.1138673

**Published:** 2023-03-09

**Authors:** Hiroshi Moriyama, Sadahiro Nomura, Hirochika Imoto, Fumiaki Oka, Yuichi Maruta, Naomasa Mori, Natsumi Fujii, Michiyasu Suzuki, Hideyuki Ishihara

**Affiliations:** ^1^ Departments of Neurosurgery, Graduate School of Medicine, Yamaguchi University, Ube, Yamaguchi, Japan; ^2^ Epilepsy Center, Yamaguchi University Hospital, Ube, Yamaguchi, Japan

**Keywords:** electrocorticograms, fast ripple, febrile seizures, hyperthermia, loss of righting reflex, transient receptor potential melastatin 8 (TRPM8), TRPM8 agonist, TRPM8 knockout mice

## Abstract

**Background:** Febrile seizures (FSs) are the most frequent type of seizures in infancy and childhood. Epileptiform discharges (EDs) on electroencephalogram at the time of first FS recurrence can increase the risk of epilepsy development. Therefore, inhibition of EDs is important. Recently, WS-3, a transient receptor potential melastatin 8 (TRPM8) agonist, reportedly suppressed penicillin G-induced cortical-focal EDs. However, the effects of TRPM8 agonists on FSs remain unknown. In this study, we aimed to clarify the effects of the TRPM8 agonist, and the absence of TRPM8 channels, on hyperthermia-induced FS by analyzing the fast ripple band.

**Methods:** Hyperthermia (43°C for 30 min) induced by a heating pad caused FSs in postnatal day 7 wild-type (WT) and TRPM8 knockout (TRPM8KO) mice. FSs were defined as EDs occurring during behavioral seizures involving hindlimb clonus and loss of the righting reflex. Mice were injected with 1% dimethyl sulfoxide or 1 mM WS-3 20 min before the onset of hyperthermia, and electroencephalograms; movies; and rectal, brain and heating pad temperatures were recorded.

**Results:** In wild-type mice, WS-3 reduced the fast ripple amplitude in the first FS without changing rectal and brain temperature thresholds. In contrast, the anti-FS effect induced by the TRPM8 agonist was not observed in TRPM8KO mice and, compared with wild-type mice, TRPM8 deficiency lowered the rectal and brain temperature thresholds for FSs, exacerbated the fast ripple amplitude, and prolonged the duration of the initial FS induced by hyperthermia.

**Conclusion:** Our findings suggest that TRPM8 agonists can be used to treat hyperthermia-induced FSs.

## Introduction

Febrile seizures (FSs) are the most frequent type of seizures in infancy and childhood, and usually occur between 3 months and 5 years of age in association with a fever (Mewasingh, 2014). The majority of FSs are simple and have a favorable prognosis, although approximately 15%–20% are complex and can increase the risk of epilepsy development (Camfield et al., 1994; Syndi Seinfeld and Pellock, 2013; Camfield and Camfield, 2015; Ram and Newton, 2015; Leung et al., 2018; Pavone et al., 2022). Another risk factor for the development of epilepsy is epileptiform discharges (EDs) on electroencephalogram (EEG) at the time of first FS recurrence (Pavlidou and Panteliadis, 2013). Thus, inhibiting EDs during FSs is important. Recently, icilin, a transient receptor potential melastatin 8 (TRPM8) agonist, was shown to suppress penicillin G-induced cortical-focal EDs (Moriyama et al., 2019). However, the effects of TRPM8 agonists on FS remain unknown.

TRPM8 is a cold receptor activated by temperatures of 10°C–26°C (McKemy et al., 2002; Bautista et al., 2007). TRPM8 is also activated by TRPM8 agonists (McKemy et al., 2002; Behrendt et al., 2004; McKemy, 2005) and is expressed in areas of the rodent brain, including the hypothalamus, hippocampus, and frontal cortex (Voronova et al., 2013; Wang et al., 2017; Ordas et al., 2019). Icilin and WS-3, which are both TRPM8 agonists, suppressed drug-induced EDs and epileptic seizures (Moriyama et al., 2019, 2021), while a TRPM8 deficit led to more severe drug-induced EDs and epileptic seizures (Moriyama et al., 2021). In addition, cold-driven TRPM8 channels are crucial for detecting warmth (Paricio-Montesinos et al., 2020).

The above results suggest that TRPM8 deficits affect FSs, but the mechanisms remain to be elucidated. Therefore, in this study, we aimed to clarify the effects of a TRPM8 agonist, and the absence of TRPM8 channels, on hyperthermia-induced FSs.

## Materials and methods

### Animals

Pregnant female C57BL/6N mice were purchased from CLEA Japan Inc. (Tokyo, Japan). Male and female TRPM8 homozygous knockout (TRPM8KO) mice were supplied by Thermal Biology Group, Exploratory Research Center on Life and Living Systems. (Okazaki, Japan). Mice were housed in cages, singly or in pairs, and maintained under standard laboratory conditions in a temperature- and humidity-controlled room (25°C ± 2°C and 55% ± 5%, respectively) under a 12-h light/dark cycle (lights on at 8:00 a.m.) (Dhaka et al., 2007). Adult mice had free access to food and water. Newborn mice were co-housed and were free to breastfeed from the dams. The animal care and experimental procedures were approved by the Experimental Animal Care and Use Committee of Yamaguchi University School of Medicine, Japan. All experiments were performed in accordance with the guidelines of the Japan Association of Laboratory Animal Facilities of National University Corporations.

### Hyperthermia-induced FS model

**FIGURE 1:**
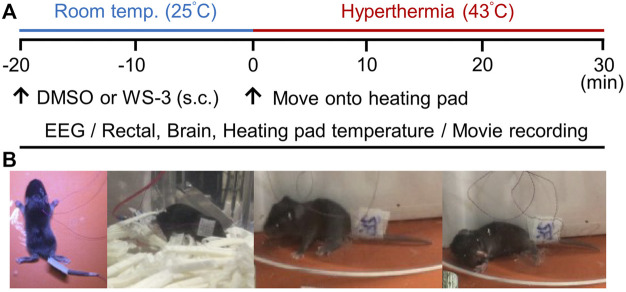
Experimental protocol for inducing febrile seizures (FSs) *via* hyperthermia. **(A)** Protocols for recording electroencephalography data and movies, measuring rectal, brain and heating pad temperatures, and analyzing the effects of transient receptor potential melastatin 8 agonist treatment. **(B)** Postoperative images of postnatal day 7 mice and a typical FS involving loss of the righting reflex caused by hyperthermia.

To determine whether TRPM8 channel deficiency exacerbates FSs in mice, we compared hyperthermia-induced seizures between wild-type (WT) and TRPM8KO mice. FSs were induced in accordance with previously reported methods (Koyama et al., 2012; Kasahara et al., 2019) with the following modifications. To minimize noise and artifacts during EEG recording, FSs were induced by exposing postnatal day (P)7 mice to hyperthermia using a heating pad. Before hyperthermia induction, the P7 mice were acclimatized to a cage at room temperature (25°C) for 20 min (Figure 1A). Hyperthermia was induced by setting the heating pad to 43°C in an acrylic cylinder (16 cm in diameter × 20 cm high). The core temperature of the mice was raised during 30 min of hyperthermia. In accordance with previous reports, with some modifications (Koyama et al., 2012; Kasahara et al., 2019), the FSs were define as epileptic discharges occurring during behavioral seizures involving the hindlimb clonus and loss of the righting reflex (Figure 1B). FSs were frequently observed during hyperthermia, as in previous reports (Koyama et al., 2012; Kasahara et al., 2019).

### Rectal and brain temperature sensor and electrode implantation

The P7 mice were anesthetized with sevoflurane (1.5% for induction and 0.5% for maintenance; Pfizer Japan, Tokyo, Japan). A thin thermocouple (IT-23; Physitemp, Tokyo, Japan) was inserted into the rectal passage and fixed to the tail using surgical tape. To maintain the body and brain temperatures during operation, the heating pad temperature was adjusted to 36.0°C ± 0.5°C to control the rectal temperature of the mice in their home cages.

After performing a scalp incision, we created two slits in the soft skull using tweezers. EEG recording and brain temperature sensors were implanted through a slit in the right sensorimotor cortex and the reference electrode was implanted through a slit in the cerebellum. After surgery, the scalps were adhered using cyanoacrylate adhesive (Tokyo Measuring Instruments Laboratory, Co., Ltd., Tokyo, Japan). A ground electrode was placed under the back skin.

### EEG recording

EEG was recorded by referring to a previously reported method (Moriyama et al., 2021). EEG recording was started immediately before the mice were moved to the cage maintained under room temperature conditions (25°C). EEG was continuously recorded for 50 min (20 min at room temperature and 30 min under hyperthermic conditions). EEG was amplified by a bio-amplifier (EX-1; Dagan Corporation, Minneapolis, MN, United States) using an analogue-to-digital converter at a sampling rate of 2 kHz (PowerLab 8/30; AD Instruments, Castle Hill, Australia). The conditions for recording EEGs were as follows: low-frequency filter, 0.1 Hz; high-frequency filter, 10 kHz; and notch filter, off.

### EEG analysis

To evaluate the effects of the TRPM8 agonist on hyperthermia-induced FSs, the EEG was Fourier-transformed. The initial FSs were analyzed to determine the effects of the TRPM8 agonist and TRPM8 deficiency on the rectal and brain temperature thresholds for FSs. We calculated the amplitude and duration of the fast ripple (250–500 Hz) during the first FS using Lab Chart Pro (ver. 8.1.21; AD Instruments) by referring to a previously reported method (Sheybani et al., 2018; Kasahara et al., 2019). Because evaluating fast ripples after resecting epileptogenic brain tissue aids prediction of seizure recurrence (Burelo et al., 2021), changes in fast ripples in EEG could serve as a pathological indicator of FSs (Wu et al., 2001; Kasahara et al., 2019) and epileptic seizures (Engel et al., 2009; Simeone et al., 2014).

### Rectal and brain temperature recording

Rectal and brain temperatures were recorded by referring to a previously reported method (Moriyama et al., 2021), and started immediately before the mice recovered from the anesthesia and were moved to the cage maintained under room temperature conditions (25°C). Both temperatures were continuously recorded for 50 min.

### Behavioral recording

Behavior was recorded using an iPad device (Apple Inc., Cupertino, CA, United States); recording began immediately before the mice were moved to a new cage maintained under room temperature conditions (25°C). Behavior was continuously recorded for 50 min.

### Drug

Vehicle (1% dimethyl sulfoxide [DMSO]; Merck KGaA, Darmstadt, Germany) was prepared using saline, and 1 mM of WS-3 (Funakoshi, Tokyo, Japan) was dissolved in the 1% DMSO by referring to a previously reported method (Moriyama et al., 2021).

### Drug treatment

To evaluate the efficacy of the TRPM8 agonist against FSs, 1 mM WS-3 (10 ml/kg) or 1% DMSO was subcutaneously (s.c.) administrated 20 min before the onset of hyperthermia. The mice were randomly assigned to the following experimental groups: S.c. injection of 1% DMSO and 1 mM WS-3 in WT mice (WT/DMSO group and WT/WS-3 group) and s.c. injection of 1% DMSO and 1 mM WS-3 in TRPM8KO mice (TRPM8KO/DMSO group and TRPM8KO/WS-3 group).

### Statistical analysis

Statistical analyses were performed using JMP Pro 16.1.0 software (SAS Institute Inc., Cary, NC, United States). All results are expressed as mean ± standard error of the mean. Statistically significant differences were evaluated by Tukey’s test with *p* < 0.05 indicating statistical significance.

## Results

### Changes of rectal and brain temperature under room temperature and hyperthermic conditions

**FIGURE 2:**
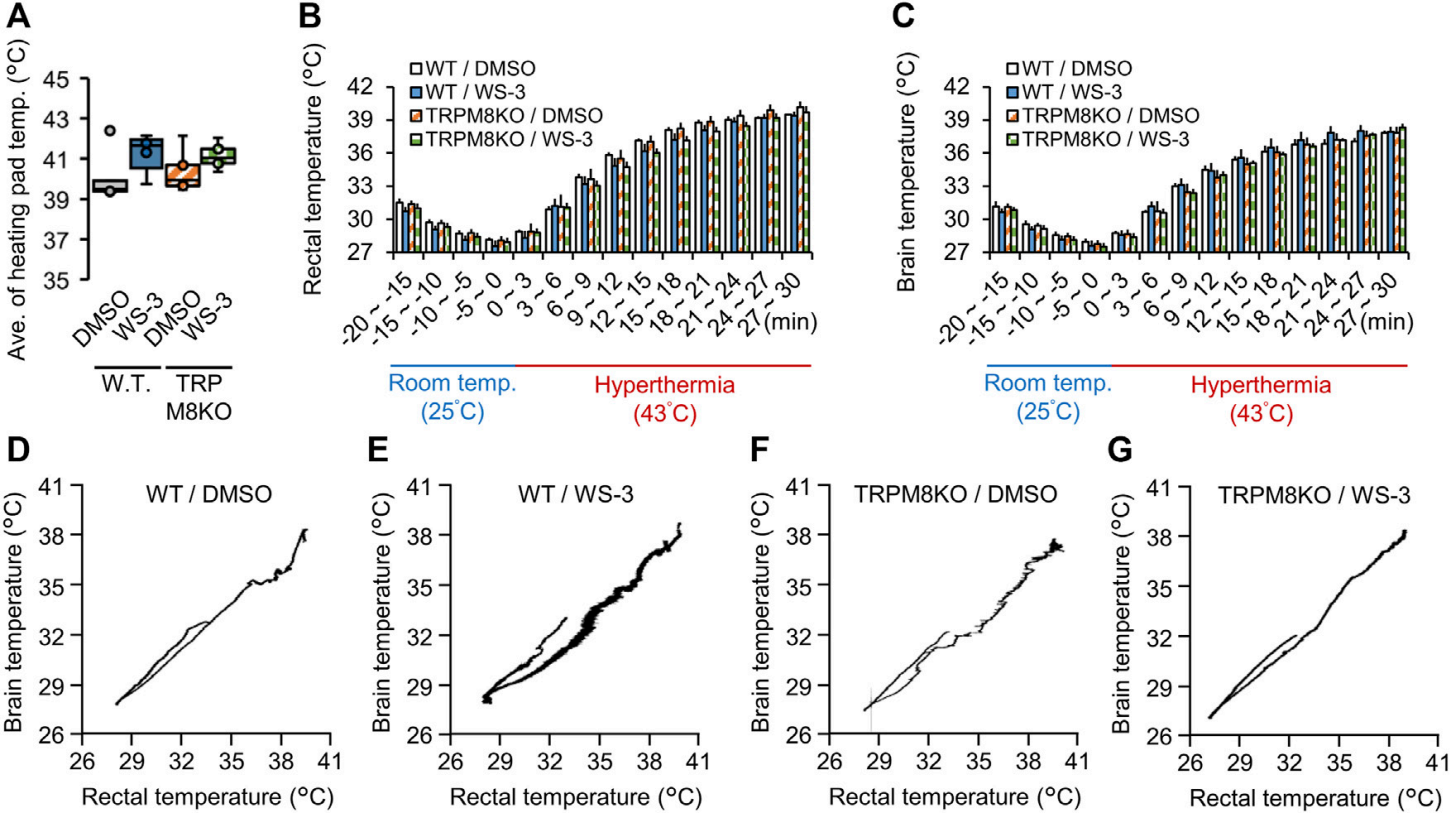
Effects of a transient receptor potential melastatin 8 (TRPM8) agonist and TRPM8 deficiency on rectal and brain temperatures under the hyperthermic condition. **(A)** The surface temperature of the heating pad under the hyperthermic condition (43°C for 30 min) in wild-type (WT) mouse/dimethyl sulfoxide (DMSO) (white: *n* = 5) and WT/WS-3 (blue: *n* = 5) groups, and TRPM8 knockout (TRPM8KO) mouse/DMSO (orange oblique line: *n* = 5) and TRPM8KO/WS-3 (green squares: *n* = 5) groups. Changes of **(B)** rectal and **(C)** brain temperature in each group under room temperature (25°C for 20 min) and hyperthermia (43°C for 30 min) conditions. **(D–G)** Representative rectal and brain temperature trends under room temperature and hyperthermic conditions in each group.

To confirm that the hyperthermic condition was identical among the groups, we measured the heating pad surface temperature in all groups. The average temperature under the hyperthermic condition was not different among the groups (WT/DMSO; 40.10°C ± 0.58°C, WT/WS-3; 41.32°C ± 0.42°C, TRPM8KO/DMSO; 40.38°C ± 0.49°C, and TRPM8KO/WS-3; 41.14°C ± 0.29°C, respectively; Figure 2A). Figures 2B, C shows the trends in rectal and brain temperatures under the hyperthermic condition (43.0°C). To clarify the effects of the TRPM8 agonist and TRPM8 deficiency on rectal and brain temperature, the changes therein were compared among the groups; there was no group difference at any timepoint (Figures 2B, C). Figures 2D–G shows representative examples of rectal and brain temperature trends under the room temperature and hyperthermic conditions in each group.

### The TRPM8 agonist WS-3 did not affect the rectal and brain temperature thresholds in WT mice

**FIGURE 3:**
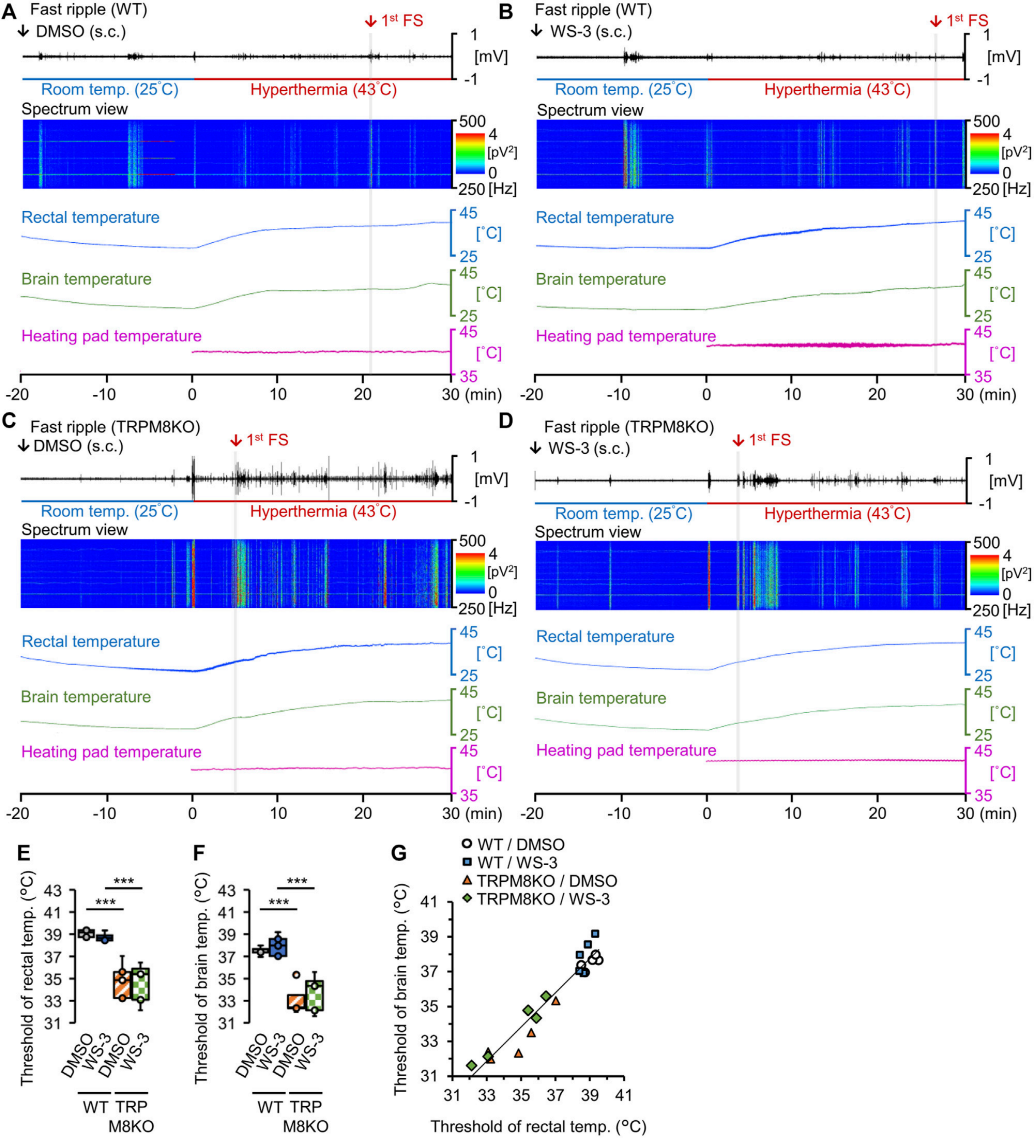
Effects of the transient receptor potential melastatin 8 (TRPM8) agonist and TRPM8 deficiency on rectal and brain temperature thresholds under the hyperthermic condition. **(A–D)** Representative trends in fast ripple amplitudes, power spectra for the fast ripple band, and rectal, brain, and heating pad temperatures in **(A)** wild-type (WT) mouse/dimethyl sulfoxide (DMSO) and **(B)** WT/WS-3 groups, and **(C)** TRPM8 knockout (TRPM8KO) mouse/**(D)** DMSO and TRPM8KO/WS-3 groups. Red arrows and gray columns indicate the time required for hyperthermia to induce the first febrile seizure (FS) in each group. Rectal **(E)** and brain **(F)** temperature thresholds for the first FS induced by hyperthermia in the WT/DMSO (white: *n* = 5) and WT/WS-3 (blue: *n* = 5) groups, and TRPM8KO/DMSO (orange oblique line: *n* = 5) and TRPM8KO/WS-3 (green squares: *n* = 5) groups. **(G)** Correlation between the rectal and brain temperature thresholds for the first FS induced by hyperthermia in each group in the WT/DMSO (white circle: *n* = 5) and WT/WS-3 (blue square: *n* = 5) groups, and TRPM8KO/DMSO (orange triangle: *n* = 5) and TRPM8KO/WS-3 (green rhombus: *n* = 5) groups.

We recorded the fast ripple amplitudes and power spectra, and the rectal, brain, and heating pad temperatures. The fast ripple band was color-coded according to intensity to clearly show the effects of the TRPM8 agonist and TRPM8 deficiency on FSs (Figures 3A–D). To reveal the effects of the TRPM8 agonist on the rectal and brain temperature thresholds for the first FS, the threshold temperatures were compared among the groups. With DMSO administration to WT mice, the rectal and brain temperature thresholds for the first FS were 39.27°C ± 0.22°C and 37.52°C ± 0.17°C, respectively (Figures 3E, F). Subcutaneous (s.c.) administration of the TRPM8 agonist did not affect the rectal or brain threshold temperature for the first FS (38.97°C ± 0.36°C and 38.05°C ± 0.50°C, *p* = 0.981 and *p* = 0.952, respectively, Tukey’s test; Figures 3E, F). The correlation coefficient between the rectal and brain temperature thresholds for the first FS was 0.936 (*n* = 20; Figure 3G).

### In WT mice, the TRPM8 agonist reduced the fast ripple amplitude during the first FS

**FIGURE 4:**
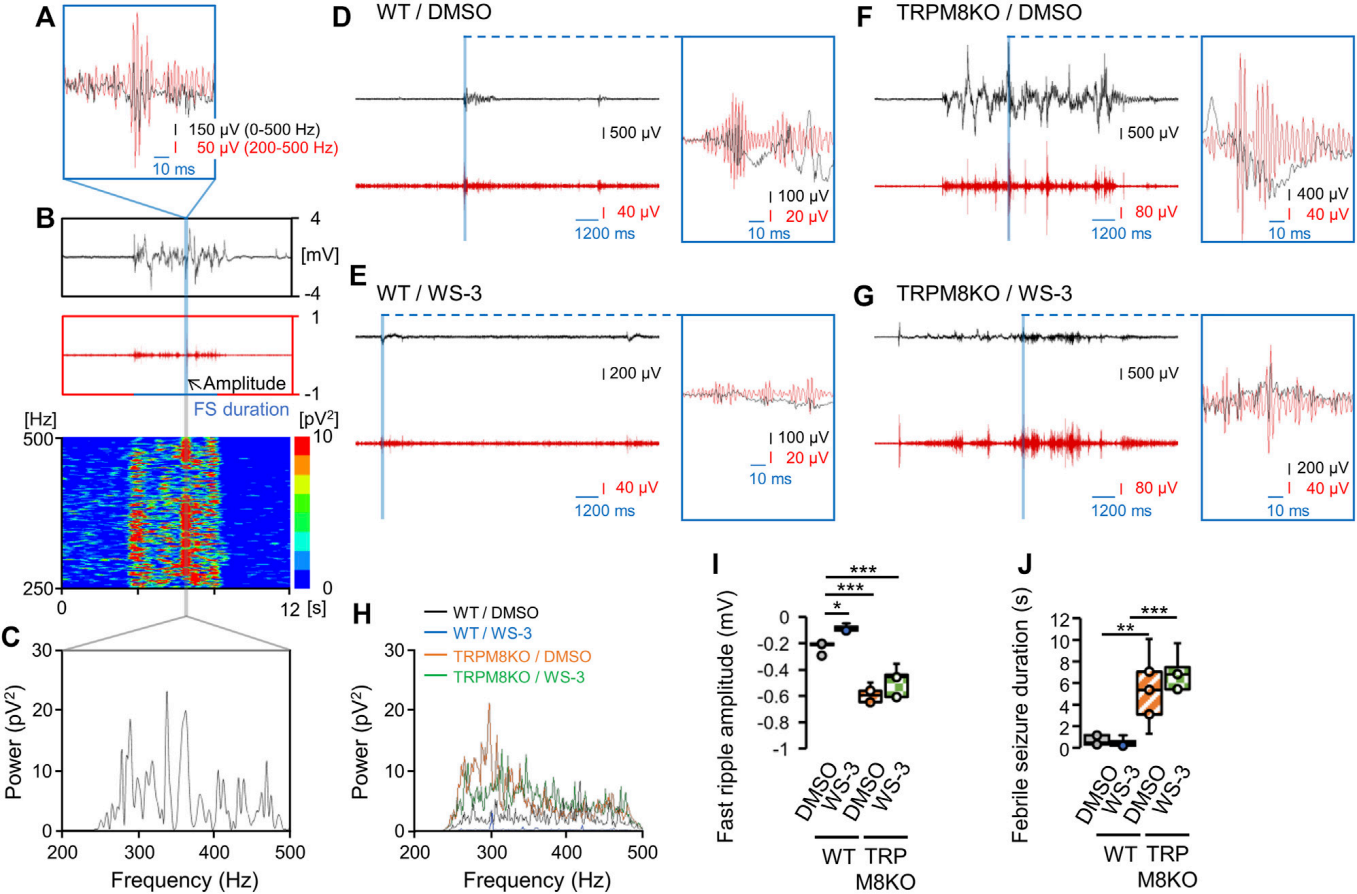
A transient receptor potential melastatin 8 (TRPM8) agonist improved hyperthermia-induced febrile seizures (FSs), whereas TRPM8 deficiency led to more severe FSs. **(A–C)** Maximum amplitude of the fast ripple and power spectrum of the fast ripple band during the first FS. **(A)** Example overlay of the EEG on a fast ripple spike. **(B)** Representative trends in the fast ripple amplitude on electroencephalogram (EEG) and fast ripple band power spectrum for the first FS. **(C)** Example power spectrum for the fast ripple band. **(D–G)** Examples of EEG and fast ripples, and of EEGs overlaid on fast ripple spikes, in each group. **(H)** Example power spectra for fast ripple bands in each group. **(I)** Maximum fast ripple amplitude for the first FS and **(J)** Duration of the first FS in wild-type (WT) mouse/dimethyl sulfoxide (DMSO) (white: *n* = 5) and WT/WS-3 (blue: *n* = 5) groups, and TRPM8 knockout (TRPM8KO) mouse/DMSO (orange oblique line: *n* = 5) and TRPM8KO/WS-3 (green squares: *n* = 5) groups. The results are shown as mean ± standard error of the mean. *p < 0.05, **p < 0.01, ***p < 0.001. All analyses were followed by Tukey’s test.

To evaluate the effects of the TRPM8 agonist on seizure activity, the amplitude and duration of the first ripple for the first FS were analyzed in WT mice (Figures 4A–J). Figures 4A–C shows the methods used to determine the maximum fast ripple amplitude and duration of the first FS, and the power spectrum of the fast ripple band. Figures 4D–G shows fast ripple spikes overlaid with EEGs in each group. The TRPM8 agonist suppressed the hyperthermia-induced hyperactivity in the fast ripple band in WT mice (Figure 4H). With DMSO administration in WT mice, the fast ripple amplitude and duration were −0.221 ± 0.018 mV and 0.32 ± 0.10 s, respectively, under the hyperthermic condition (Figures 4I, J). Pre-administration of the TRPM8 agonist significantly reduced the fast ripple amplitude, but not the fast ripple duration (−0.085 ± 0.011 mV and 0.24 ± 0.09 s, *p* = 0.029 and *p* = 0.999, respectively, Tukey’s test; Figures 4I, J).

### A lack of TRPM8 channels reduced the FS thresholds

To reveal the effects of TRPM8 deficiency on the rectal and brain temperature thresholds for the first FS, the thresholds were compared between WT and TRPM8KO mice. Under the hyperthermic condition, TRPM8 deficiency reduced both thresholds (34.76°C ± 0.74°C and 33.11°C ± 0.61°C, respectively, both *p* < 0.001, Tukey’s test; Figures 3E, F).

To evaluate the effects of TRPM8 deficiency on seizure activity, the amplitude and duration of the first ripple for the first FS were compared between WT and TRPM8KO mice. Compared with WT mice, TRPM8 deficiency exacerbated the hyperthermia-induced hyperactivity in the fast ripple band (Figure 4H). In TRPM8KO mice, the TRPM8 agonist did not suppress the exacerbated hyperactivity in the fast ripple band (Figure 4H). TRPM8 deficiency led to a greater increase in fast ripple amplitude and duration for the first FS induced by hyperthermia compared with WT mice (TRPM8KO/DMSO; −0.589 ± 0.028 mV and 2.69 ± 0.77 s, *p* < 0.001 and *p* = 0.007, respectively, Tukey’s test; Figures 4I, J). In TRPM8KO mice, the TRPM8 agonist did not suppress the increase in fast ripple amplitude (−0.493 ± 0.050 mV, *p* = 0.164, Tukey’s test; Figure 4I). TRPM8 was also associated with a prolonged fast ripple duration even with pre-administration of the TRPM8 agonist (3.47 ± 0.40 s, *p* = 0.594, Tukey’s test; Figure 4J).

## Discussion

This study compared changes in rectal and brain temperatures, and FS severity, between WT mice and mice lacking TRPM8 channels, and also investigated the effects of a TRPM8 agonist on FS. There were three major findings. First, the changes of rectal and brain temperature caused by hyperthermia treatment were not different between the WT and TRPM8KO mice. Second, TRPM8 deficiency lowered the rectal and brain temperature thresholds for the first FS induced by hyperthermia, independent of the changes in those temperatures. Third, in WT mice, TRPM8 agonist administration before hyperthermia resulted in lower-amplitude abnormal discharges. In contrast, the anti-FS effect induced by the TRPM8 agonist was not observed in TRPM8KO mice, and TRPM8 deficiency reduced the fast ripple amplitude, and prolonged its duration, for the first FS induced by hyperthermia compared with WT mice.

Our data indicate that the TRPM8 agonist reduced the fast ripple amplitude for abnormal discharges induced by hyperthermia (43°C) (Figure 4I). This result agrees with previous studies in which a TRPM8 agonist suppressed induced epileptic discharges (Moriyama et al., 2019, 2021). This reduction of the fast ripple amplitude of abnormal discharges during FS was not observed in our TRPM8KO mice even when the TRPM8 agonist was administrated before the heat load test at 43°C (Figure 4I). Our result was supported by a previous report, in which anti-epileptic effects of the TRPM8 agonist in TRPM8KO mice were not detected even when the TRPM8 agonist was administered to the epileptic focus (Moriyama et al., 2021). The present data indicate that TRPM8 agonists can inhibit excessive excitability in neurons, but the mechanisms underlying the modulatory action of TRPM8 agonists on anti-FS effects remain to be elucidated. The mechanisms through which TRPM8 agonists affect abnormal discharges also remain controversial. In the peripheral nervous system, menthol and icilin increased the miniature excitatory postsynaptic current (EPSC) frequency (Baccei et al., 2003; Wrigley et al., 2009; Kumamoto et al., 2014), and these TRPM8 agonists both enhanced glutamatergic neuronal transmission. In contrast, TRPM8 agonists reduced cellular excitability in other reports. A low concentration of icilin (3 mmol·L^−1^) decreased the amplitude of evoked EPSCs in 23% of lamina I and II dorsal horn neurons (Wrigley et al., 2009). Menthol reduces the excitation of rat hippocampal neurons in culture and suppresses the epileptic activity induced by pentylenetetrazole injection and electrical kindling *in vivo* (Zhang et al., 2008). These conflicting results regarding the effects of TRPM8 agonists may depend on the pathways through which TRPM8 agonists affect the excitability of neurons. Indeed, menthol enhanced currents induced by low concentrations of gamma-aminobutyric acid (GABA) and directly activated the GABAA receptor in hippocampal neurons in culture (Zhang et al., 2008).

To elucidate the effects of an absence of TRPM8 channels on hyperthermia-induced FS, we compared the rectal and brain temperature thresholds, and the fast ripple amplitude and duration on EEG, between WT and TRPM8KO mice. TRPM8 deficiency reduced the rectal and brain temperature thresholds for the first FS, reduced the fast ripple amplitude of EDs, and lengthened the fast ripple duration (Figures 3E, F, 4I, J); these results indicate that FSs are worsened by TRPM8 deficits. Our findings are supported by Moriyama et al. (2021), who found that epileptic discharges and seizures were exacerbated in TRPM8KO mice. Because the distribution of TRPM8 channels in the rodent brain was reported relatively recently (Ordas et al., 2019), the mechanisms through which TRPM8 deficits worsen FSs remain unknown. Thus, further studies are required to determine how TRPM deficiency promotes the development of FSs.

To clarify whether TRPM8 deficits affect heat dissipation and storage in the heat load test, we evaluated changes in heating pad, rectal, and brain temperatures in TRPM8KO mice during hyperthermia. Our data showed that the changes of rectal and body temperature under hyperthermia (43°C) were not different between WT and TRPM8KO mice (Figures 2B, C). The relative lack of effect of TRPM8 deficiency on the changes of rectal temperature induced by hyperthermia may be explained by TRPM8 being activated by temperatures of 8°C–28°C (McKemy et al., 2002; Peier, 2002). Under an environmental temperature at 29°C, changes in body temperature were not different between WT and TRPM8KO adult mice, whereas the core body temperature decreased more in TRPM8KO mice in a cold environment (17°C) (Reimúndez et al., 2018). Cold-sensitive C-fibers are predominantly TRPM8+ (Bautista et al., 2007; Dhaka et al., 2008) and loss of the cold-driven TRPM8 channel abolished the ability to detect warmth (Paricio-Montesinos et al., 2020); in line with this, TRPM8-deficient mice showed reduced avoidance of noxious cold temperatures (Dhaka et al., 2007).

We also found that s.c. injection of WS-3, a selective TRPM8 agonist, did not affect the changes of rectal and brain temperatures induced by the heat load test conducted at 43°C in WT or TRPM8KO mice (Figures 2B, C). Our results suggest that the changes of heat dissipation and storage associated with TRPM8 deficiency do not worsen FSs. However, in a previous report, a single application of the TRPM8 agonist menthol to the human skin surface led to heat storage enhancement, which was mediated by a vasoconstrictor response (Gillis et al., 2015). Icilin, which is also a TRPM8 agonist, produced a significant increase of body temperature at a dose of 8.2 mg/kg, whereas IGM-18, a TRPM8 antagonist, produced a significant decrease at the highest dose (10 mg/kg) (De Caro et al., 2019). These conflicting results may be mainly explained by the drastic increase in rectal and brain temperatures induced by the 43°C heat load test masking the effect of the TRPM8 agonist on core body temperature. Our data showed that the heat load test (43°C for 30 min) increased the rectal temperature by 11.11°C (from 28.46°C to 39.57°C) in WT mice following WS-3 administration (Figure 2B), whereas the rectal temperature did not increase 30 min after icilin (7.5 mg/kg) administration (De Caro et al., 2019). Taken together, our data suggest that FSs were rarely improved by TRPM8 agonists through the regulation of rectal and brain temperature; the improvements were mainly driven by suppression of the excessive neuronal excitability induced by hyperthermia.

In summary, we compared changes in rectal and brain temperature, and the effects of a TRPM8 agonist on FSs, between WT and TRPM8KO mice. Our results showed that the FS-suppressing effects of the TRPM8 agonist rarely involved modulation of the rectal and brain temperatures. In addition, TRPM8 deficits did not affect the changes of rectal and brain temperature, but did reduce the latency to the initial FS and increased seizure severity. These results indicate that FSs are improved by TRPM8 agonists mainly *via* suppression of the excessive neuronal excitability induced by hyperthermia. In conclusion, TRPM8 agonists may be a new treatment option for patients with hyperthermia-induced FS.

## Data Availability

The original contributions presented in the study are included in the article/supplementary material, further inquiries can be directed to the corresponding author.

## Abbreviations

TRPM8, transient receptor potential melastatin 8; WT, wild-type; FS, febrile seizure; DMSO, dimethyl sulfoxide; TRPM8KO, TRPM8 knockout; EEG, electroencephalogram; ED, epileptiform discharge; s.c., subcutaneous; EPSC, excitatory postsynaptic current; GABA, gamma-aminobutyric acid; P, postnatal day.
